# CHD1L contributes to cisplatin resistance by upregulating the ABCB1–NF-κB axis in human non-small-cell lung cancer

**DOI:** 10.1038/s41419-019-1371-1

**Published:** 2019-02-04

**Authors:** Yang Li, Li-Ru He, Ying Gao, Ning-Ning Zhou, Yurong Liu, Xin-Ke Zhou, Ji-Fang Liu, Xin-Yuan Guan, Ning-Fang Ma, Dan Xie

**Affiliations:** 1 Affiliated Cancer Hospital & Institute of Guangzhou Medical University; Laboratory of Protein Modification and Degradation, State Key Laboratory of Respiratory Disease, Guangzhou Medical University, Guangzhou, Guangdong 511436 China; 2 State Key Laboratory of Oncology in South China; Collaborative Innovation Center for Cancer Medicine, Sun Yat-Sen University Cancer Center, Guangzhou, Guangdong China; 3 Department of Oncology, The First Affiliated Hospital, Sun Yat-Sen University, Guangzhou, Guangdong China; 4 The Fifth Affiliated Hospital of Guangzhou Medical University, Guangzhou, China

## Abstract

Chromodomain helicase/ATPase DNA binding protein 1-like gene (*CHD1L*) is a recently identified gene associated with malignant tumor progression and patient chemotherapy resistance in human hepatocellular carcinoma (HCC). Previously, we found an association between CHD1L overexpression and poor patient survival in non-small-cell lung cancer (NSCLC). However, little is known about the relationship between CHD1L expression and chemotherapy resistance of NSCLC. By employing immunohistochemistry, we analyzed the expression of CHD1L in NSCLC samples and elucidated the roles and mechanism of CHD1L in NSCLC chemoresistance. We found that the increased expression of CHD1L is positively correlated with a shorter survival time of patients who had received chemotherapy after surgery. We also found that the expression of CHD1L was increased after cisplatin treatment in A549 cells. Conversely, the depletion of CHD1L in cisplatin-resistance cells increased the cell sensitivity to cisplatin, indicating that CHD1L plays a critical role in cisplatin resistance of NSCLC cells. Importantly, we identified the ATP-Binding Cassette Sub-Family B Member (*ABCB1*) gene as a potential downstream target of CHD1L in NSCLC cells. Knocking down ABCB1 coupled with ectopic expression of CHD1L enhanced the effect of cisplatin on NSCLC cells apoptosis. In addition, overexpressed CHD1L increase the transcription of c-Jun which targeted directly to the promoter of ABCB1. Our data demonstrate that CHD1L could induce cisplatin resistance in NSCLC via c-Jun-ABCB1–NF-κB axis, and may serve as a novel predictive marker and the potential therapeutic target for cisplatin resistance in NSCLC.

## Introduction

Lung cancer is the leading cause of cancer-related mortality worldwide^1^. Non-small-cell lung cancer (NSCLC) consists of a largely heterogeneous group of malignancies and is estimated to account for nearly 85% of all diagnosed lung cancers^2^. Cisplatin (DDP)-based chemotherapy is considered as one of the primary standard systematic treatment choices for advanced NSCLC^2–4^. Unfortunately, following cisplatin treatment a high rate of relapse occurs despite so many efforts on overcoming drug resistance^5^. Therefore, a better understanding of the molecular mechanisms underlying cisplatin resistance is crucial for optimizing the individual therapies and achieve better survival outcomes for NSCLC patients^6^.

Chromodomain helicase/ATPase DNA binding protein 1-like gene (*CHD1L*) is a newly identified oncogene isolated from a region of chromosome 1q that is frequently amplified in human hepatocellular carcinoma (HCC)^7^. Our previous work has demonstrated that CHD1L contributes to HCC cell migration, invasion, and metastasis. We also found that CHD1L expression is positively associated with tumor progression in HCC patients^8–11^. Recently, CHD1L has also been reported as a novel biomarker for patients’ prognosis in several types of malignant tumors including breast cancer^12^, gastric cancer^13^, colorectal cancer^14^, bladder cancer^15^ as well as ovarian cancer^16^. Our previous study showed that overexpression of CHD1L associated with the advanced diagnostic stage and poorer survival rate of NSCLC patients^17^. However, little is known about the relationship between CHD1L expression and chemotherapy resistance of NSCLC.

In this study, high expression of CHD1L was detected in NSCLC patients tissues and the overexpressed of CHD1L was associated with poorer survival rates in NSCLC patients who were treated with cisplatin-based chemotherapy. We also investigated the role of CHD1L in NSCLC cell-cisplatin resistance by analyzing CHD1L function both in vitro and in vivo. Here we demonstrate for the first time that CHD1L can contribute to cisplatin resistance by upregulating the ATP-Binding Cassette Sub-Family B Member (*ABCB1*) gene in lung cancer cells. Upregulation of ABCB1 by CHD1L is dependent on c-Jun transcription in NSCLC cells. Moreover, NF-κB pathway is closely correlated with ABCB1 expression. Our results provide the functional and mechanistic links between CHD1L expression and cisplatin resistance, and therefore indicated a potential therapeutic target for NSCLC.

## Materials and methods

### Patients and tissue specimens

Paraffin-embedded tissue samples from 248 NSCLC patients who had undergone surgery were obtained from the Pathology Department of Cancer Center, Sun Yat-sen University, Guangzhou, China, between February 1994 and January 1998. Adjuvant chemotherapy become a standard approach only after 2004, therefore adjuvant chemotherapy using cisplatin-based combinations was only administered to 89 of the patients with stage III NSCLCs. In addition to above, paraffin-embedded biopsy specimens from 30 locally advanced NSCLC patients who had received neoadjuvant cisplatin-based chemotherapy before surgery were also obtained from the archives of the Department of Pathology of Sun Yat-sen University between July 2006 and June 2012.

Data regarding cancer stage was determined according to the pathology Tumor-Node-Metastasis (pTNM) system (AJCC/UICC 2015). Tumor differentiation and histotype were determined according to the World Health Organization classification for NSCLC. To evaluate the patients’ response to neoadjuvant chemotherapy, we used the RECIST Criteria (v.1.1). This study was approved by the medical ethics committee of Cancer Center, Sun Yat-Sen University and was performed in accordance with the Declaration of Helsinki.

### Construction of tissue microarrays

The tissue microarray (TMA) was constructed according to a method described previously. Briefly, formalin-fixed, paraffin-embedded tissue blocks and their corresponding histological H&E stained slides were overlaid for tissue TMA sampling. Tissues (248 surgical resected NSCLCs) were sampled using a tissue arraying instrument (Beecher Instruments, Silver Spring, MD); and a 0.6-mm-diameter cylinder of tissue was removed from each sample. Subsequently, tissue cylinders were re-embedded into a predetermined position in a recipient paraffin block. Three cores of tissue sample were selected from each primary NSCLC and normal lung tissue, and multiple sections (5 µm thick) were cut from the TMA block and mounted on to microscope slides.

### Immunohistochemistry

The immunohistochemistry (IHC) staining of CHD1L was accomplished using a standard streptavidin-peroxidase method as previously described. The TMA sections and tissue slides were first deparaffinized and rehydrated. Any endogenous peroxidase activity was blocked with 3% H_2_O_2_ for 10 min. Next, slides were immersed in 10 mM citrate buffer (pH 6.0) and boiled in a microwave oven for 15 min for antigen retrieval. Non-specific antibody binding was blocked with 5% normal goat serum for 10 min. Slides were incubated in monoclonal antibody against CHD1L (Abcam; 1:100) at 4 °C overnight in a moist chamber. The slides were then sequentially incubated with biotinylated goat anti-mouse IgG (Santa Cruz Biotechnology; 1:100) and streptavidin-peroxidase conjugate, each for 30 min at room temperature. For a negative control an isotope-matched human IgG was used. Finally, the 3, 5-diaminobenzidine (DAB) Substrate Kit (Dako) was used for color development followed by Mayer hematoxylin counterstaining.

Expression of CHD1L primarily appeared in NSCLC nuclear. For the evaluation of CHD1L staining, a semi-quantitative scoring criterion was used in which both staining intensity and the percentage of positive cells was recorded. A staining index (ranging from 0 to 12) was determined by the intensity of CHDIL staining (0 = negative, 1 = weakly positive, 2 = moderate positive, 3 = strongly positive) multiplied by the proportion of immunopositive tumor cells (0% = 0, <10% = 1, 10% to <50% = 2, 50% to <75% = 3, ≥75% = 4). A score of 3 or higher was classified as overexpression of CHD1L. A minimum of 300 epithelial cells were counted for each sample. For scoring, two independent pathologists (Dr. Xie D and Chen JW) were blinded to the clinicopathologic information. Any inter-observer disagreements (about 5% of the total informative cases) were reviewed a second time, followed by a conclusive judgment by both pathologists.

### Cell culture and reagents

This study used human lung cancer cell lines GCL-82, PC9, A549, SPA-A1, H322 and L-78, which were purchased from China Center for Type Culture Collection (CCTCC, Shanghai, China), where all cell lines are authenticated by STR profiling before distribution. All cells were maintained in RPMI-1640 medium supplemented with 10% fetal bovine serum (FBS, Gibco, USA), 100 units/mL penicillin, and 100 mg/mL streptomycin and kept in a humidified atmosphere containing 5% CO_2_ at 37 °C. To generate cisplatin-resistant PC9 cell lines, PC9 cells were first treated with 0.25 μM of cisplatin (DDP, Sigma), and then with increased concentrations of cisplatin in a stepwise manner during with each subsequent treatment. To maintain the drug-resistant phenotype, DDP (with final concentration of 2 μM) was added to the culture media for A549/DDP cells and PC9/DDP cells. All DMSO was purchased from Sigma-Aldrich (St. Louis, MO, USA) and Opti-MEM from Gibco (Thermo Fisher Scientific, Grand Island, NY, USA).

### Western blot analysis

Whole cell lysates of lung cancer samples were prepared with a proteinase and phosphatase inhibitor cocktail (Roche, CA, USA). Protein concentrations of lysates were measured using the BCA method (Thermo Fisher Scientific, USA). 20 µg of extracted protein was loaded on to a 10% SDS-polyacrylamide gels and then transferred onto PVDF membranes (Millipore, USA). Non-specific binding was blocked on the PVDF membranes with PBS buffer containing 0.1% Tween-20, 2% BSA, and 5% nonfat dry milk. Blots were then incubated with anti-rabbit primary antibodies overnight at 4 °C. The next day, membranes were extensively washed, and then incubated with horseradish peroxidase-conjugated anti-goat (Proteintech) or anti-rabbit (Proteintech) IgG at room temperature for 1 h. Protein blots were probed with primary antibodies against CHD1L (Abcam#ab197019), p65 (Cell Signaling Technology #8242), p65-pSer536 (Cell Signaling Technology #3033), IκBα (Cell Signaling Technology#4814), and IκBα-pSer32 (Cell Signaling Technology #2859). Signals were visualized by chemiluminescence (Bio-rad, Hercules, California) and quantitated using a Quantity One system (Bio-Rad, Hercules, CA, USA).

### Construction of the recombinant lentiviral vector

The CHD1L shRNA control vector (HSH022909LVRH6GP) was constructed by and purchased from the GeneCopoeia Company (Rockville, MD, USA). The pEZ-Lv208-CHD1L and Lv208CT control vectors were also purchased from GeneCopoeia (Rockville, MD, USA). Vectors were packaged in 293FT cells using ViraPower Mix. After culturing period of 48 h, supernatant lentiviral particles were filtered by centrifugation at 500–600 × g for 10 min and then transfected into lung cancer cells. Stably infected cells were selected using puromycin (Gibco, USA). The scramble small-interfering RNA (NC) and the siRNAs targeting CHD1L(#1, 5’-CAACTTACA TATACTACTT-3’; #2, 5’-GTTCATCTTCCACGAATTG-3’) were synthesized and purchased from Gene-Pharma (Suzhou, Jiangsu, China).

### Cell counting Kit-8 (CCK8) assay

Between 1000-2000 cells were seeded in 96-well plates, and CCK8 (Beyotime Technology, Shanghai, China) was added to each well and incubated for 2 h at 37 °C. The optical density (OD) of the cultures was measured at wavelengths of 450 nm using a V Max kinetic microplate reader at different time periods. Each experiment was performed in triplicate.

### Cell apoptosis analysis

Annexin-V/PI was applied to quantify the amount of apoptotic cell. For the Annexin-V/PI binding assay, A549, A549/DDP and PC9DDP cells were exposed to 10 μM cisplatin for 24 h and transiently transfected with siRNAs. Both floating and adherent cells were collected by centrifugation and trypsinization, respectively. Cells were then re-suspended in binding buffer and subsequently stained with FITC-Annexin-V and propidium iodide (PI) (BestBio, Shanghai, China). Stained cells were quantified by using FACS under the flow cytometer (BD Biosciences, Franklin lakes, NJ, USA) according to the manufacturer’s directions.

### Animal experiments

All animal experiments were conducted according to standards regarding the use of laboratory animals and all experiments were approved by the Sun Yat-sen University Cancer Center Institutional Animal Care and Usage Committee. Female BALB/c nude mice (4–5 weeks old) were randomly divided into three groups and subcutaneously injected with A549 cells stably expressing CHD1L shRNA1, shRNA2 or a scramble shRNA. Mice were subsequently monitored for xenograft development every 3 days. When the tumors reached a diameter of 5 mm in size, the mice in each group were randomly further divided into two subgroups and treated with intraperitoneal injections of cisplatin (3 mg/kg) or an equal volume (100 μl) of normal saline (NS) every 2 days for approximately 2 weeks. Mice were sacrificed 4 weeks after cell injection, and all tumors were removed and weighed.

### Statistical analysis

Experiments were repeated at least three times for all in vitro experiments and twice for all animal experiments. All data were analyzed with SPSS 16.0 (SPSS, Chicago, IL, USA). The association of CHD1L expression with NSCLC patients’ response to chemotherapy was assessed by the Chi-square test. The overall survival rate of patients was defined as the time from the day of diagnosis to patient death. Survival curves were assessed by Kaplan–Meier method and compared by the log-rank test. *P* value <0.05 was considered significant.

## Results

### CHD1L overexpression correlates with worse outcome in cisplatin-treated advanced NSCLC patients

**Fig. 1:**
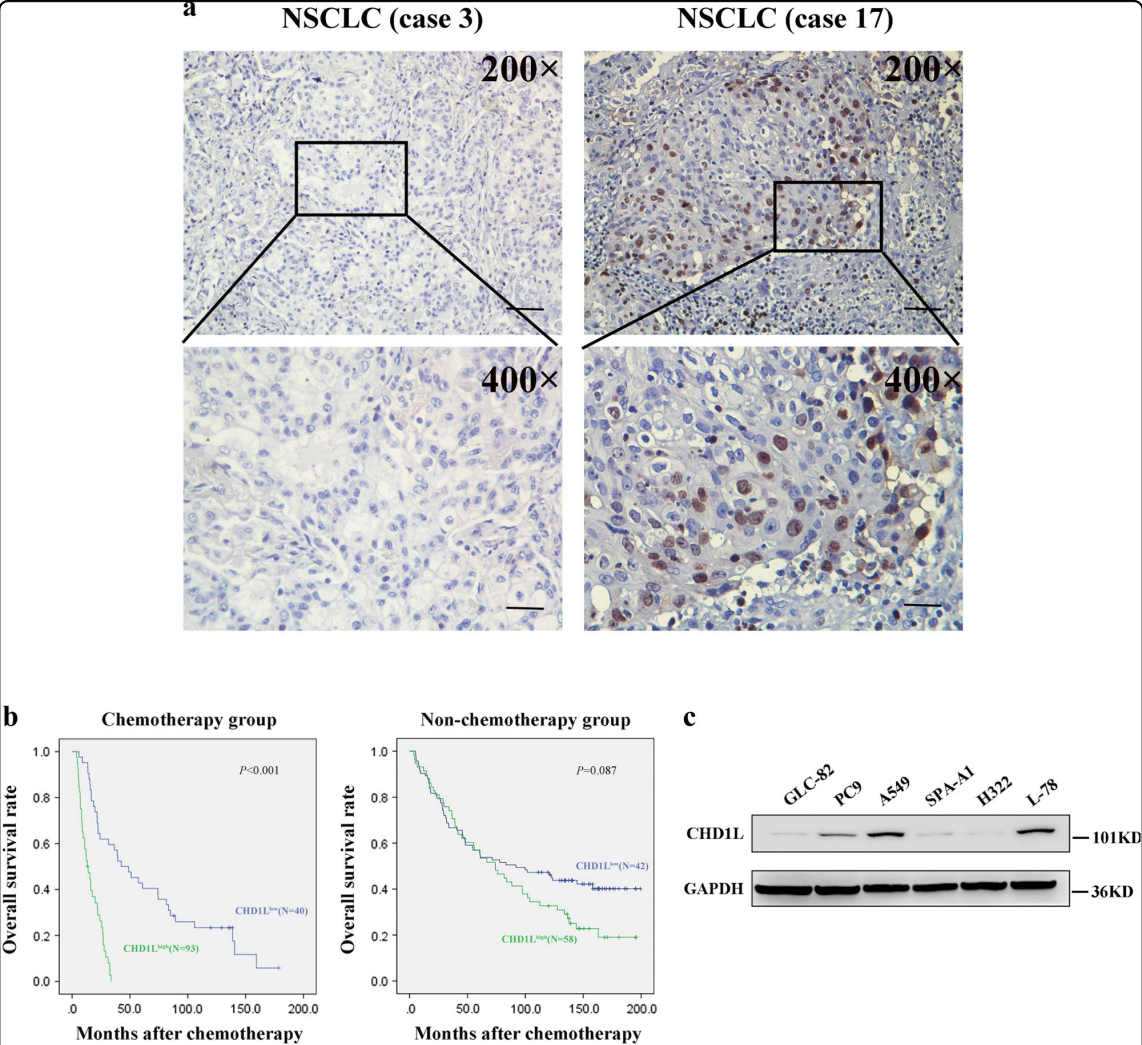
**CHD1L overexpression correlates with worse outcome in cisplatin-treated advanced NSCLC patients and expression of CHD1L in NSCLC cell lines.** **a** Immunohistochemistry showing positive nucleus staining in NSCLC patients for CHD1L, weak staining (left, original magnification, ×200); vs. strong staining (right, original magnification, ×200). The boxed regions are magnified and shown in the panel below (original magnification, ×400); scale bar:10 μm. **b** High CHD1L expression is correlated with poorer disease-free and overall survival rate in NSCLC patients (*P*<0.001). **c** The levels of CHD1L protein examined by western blotting in six different lung cancer cell lines

We examined the expression of CHD1L using IHC in 233/248 (93.9%) of our NSCLC samples. The samples that were not analyzed and therefore not included in our data compilation included unrepresentative samples, samples with too few tumor cells (<300 cells per case) and lost samples. Overexpression of CHD1L was observed in 58 (38.4%) of the 151 NSCLC patients who went without cisplatin-based adjuvant chemotherapy and 40 (48.8%) of the 82 patients who had underwent with adjuvant chemotherapy (Fig. 1a). The median survival rate was significantly shorter in patients who demonstrated CHD1L overexpression than those with normal CHD1L expression in the chemotherapy group (*P* < 0.001 log-rank test), but not in the non-chemotherapy group (*P* = 0.087, log-rank test) (Fig. 1b). In order to evaluate the association between CHD1L overexpression and the response to cisplatin-based chemotherapy in NSCLC patients, we further tested CHD1L expression by IHC in a restricted cohort of locally advanced NSCLC treated with cisplatin-based neoadjuvant chemotherapy (*n* = 30). Of the 30 patients, 12 achieved a partial response (PR), whereas the other 18 patients were evaluated as a non-response (as either no change (NC) or as progressive disease (PD)). Our data indicate a significant association between CHD1L overexpression and chemotherapy response (*P* = 0.011, Table 1).

**Table 1:** Correlation between the expression of CHD1L and therapy response in NSCLC patients (*N* = 30)

		CHD1L expression level		
	Cases	Normal expression	Overexpression	*P* value
Chemotherapy response				
NC + PD	18	5 (27.8%)	13(72.2%)	
PR	12	9 (75.0%)	3 (25.0%)	*P* < 0.05

*NC* no change, *PD* progressive disease, *PR* partial response

### CHD1L suppresses cisplatin-induced apoptosis in NSCLC cells

**Fig. 2:**
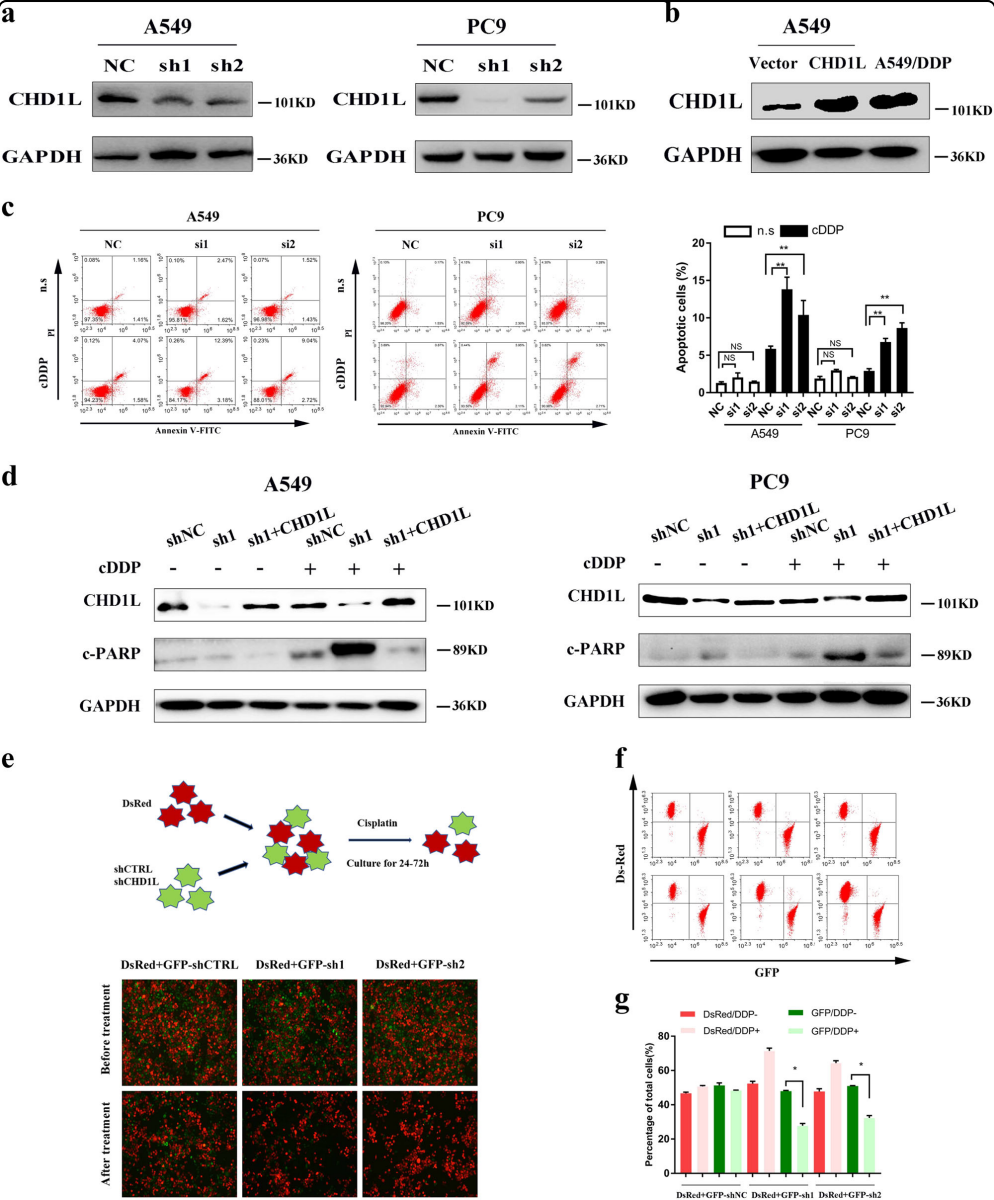
**CHD1L suppresses cisplatin-induced apoptosis in NSCLC cells.** **a** Western blotting reveals that CHD1L was efficiently knocked down by the treatment of CHD1L-shRNA-1 or CHD1L-shRNA-2 in A549 and PC9 cells. **b** Protein expression of CHD1L in vector, CHD1L-overexpressed (CHD1L) and A549/DDP cells. **c** Annexin-V-FITC/PI dual staining assay (left, representative plots for flow cytometry; right, bar charts indicating the average percentages of apoptotic cells). **d** WB assays (c-PARP, cleaved PARP; GAPDH, a loading control). **e** A549 cells stably transfected with shRNAs specifically targeting CHD1L or control shRNAs were labeled with GFP; meanwhile, the control parental cells were labeled with DsRed. GFP positive and DsRed positive cells were mixed together and subjected to cisplatin treatment (10μM) for 24-72 hours. **f** Fluorescent images of A549-shCTR-GFP and A549-shCHD1L-GFP cells mixed with A549-DsRed cells before and after cisplatin treatment. **g** Statistical analysis of flow cytometry data. NC, negative control siRNA; si1, si2, CHD1L siRNAs; Vec, empty vector transfected; CHD1L, CHD1L overexpression; n.s, normal saline. *P<0.05; **P<0.01; NS, no significance

CHD1L expression was examined in six different lung cancer cell lines by immunoblotting. The endogenous expression of CHD1L was detected in three of the cell lines (i.e., A549, PC9 and L-78), whereas the other three lines (i.e., GLC-82, SPA-A1 and H322) showed undetectable or very low levels of endogenous CHD1L (Fig. 1c). To further explore the roles of CHD1L in NSCLC, we established CHD1L downregulated NSCLC cell lines by using CHD1L shRNA transfection (the cells indicated as A549-shCHD1L and PC9-shCHD1L) (Fig. 2a). We also constructed an ectopic CHD1L overexpression A549 cell line (Fig. 2b) as well as the cisplatin-treated A549- CHD1L cells (Supplementary Fig. 1a). The Annexin-V and propidium iodide (PI) staining based FlowCytometry analysis revealed that the downregulation of CHD1L significantly enhanced cisplatin-induced apoptosis in both A549 and PC9 cells (*P* *<* 0.05 or *P* < 0.01) (Fig. 2c). Poly (ADP-ribose) polymerase (PARP) cleavage is a common marker of cell apoptosis, we therefore verified the effect of CHD1L on cisplatin-induced PARP cleavage in NSCLC cells. The result showed that knocking down of CHD1L sensitized cisplatin-induced apoptosis in A549, whereas the enforced CHD1L expression reduced PARP cleavage in cisplatin-treated A549-CHD1L cells when compared with their respective control cells (Supplementary Fig. 1b). The rescue assay showed that the enforced expression of CHD1L in A549-shCHD1L or PC9-shCHD1L cells significantly rescued cisplatin-induced apoptosis (Fig. 2d). To further determine whether CHD1L affects the sensitivity of NSCLC cells to cisplatin, the A549 cells were stably transfected with either a scramble shRNA(as a control) or shRNA specifically targeting CHD1L, and the co-transfected GFP was used as a transfection indicater, in which the parental A549 cells could be labeled with DsRed. All the GFP-labeled, CHD1LshRNA-transfected cells or the DsRed-labeled parental cells were mixed together and treated with cisplatin (Fig. 2e). After treating with cisplatin, the mixed cells were analyzed by using fluorescent microscopy or flow cytometry to detect the cells’ sensitivity to cisplatin. The results indicated that DsRed^+^ cells significantly increase after treating with cisplatin, the decrease of GFP^+^ cells was seen in shCHD1L-transfected group. As expected, neither the DsRed^+^ nor GFP^+^ cell number changed in the control shRNA-transfected group (Fig. 2f, g) (*P* *<* 0.05 or *P* < 0.01).

### Cisplatin resistance is associated with CHD1L activation

**Fig. 3:**
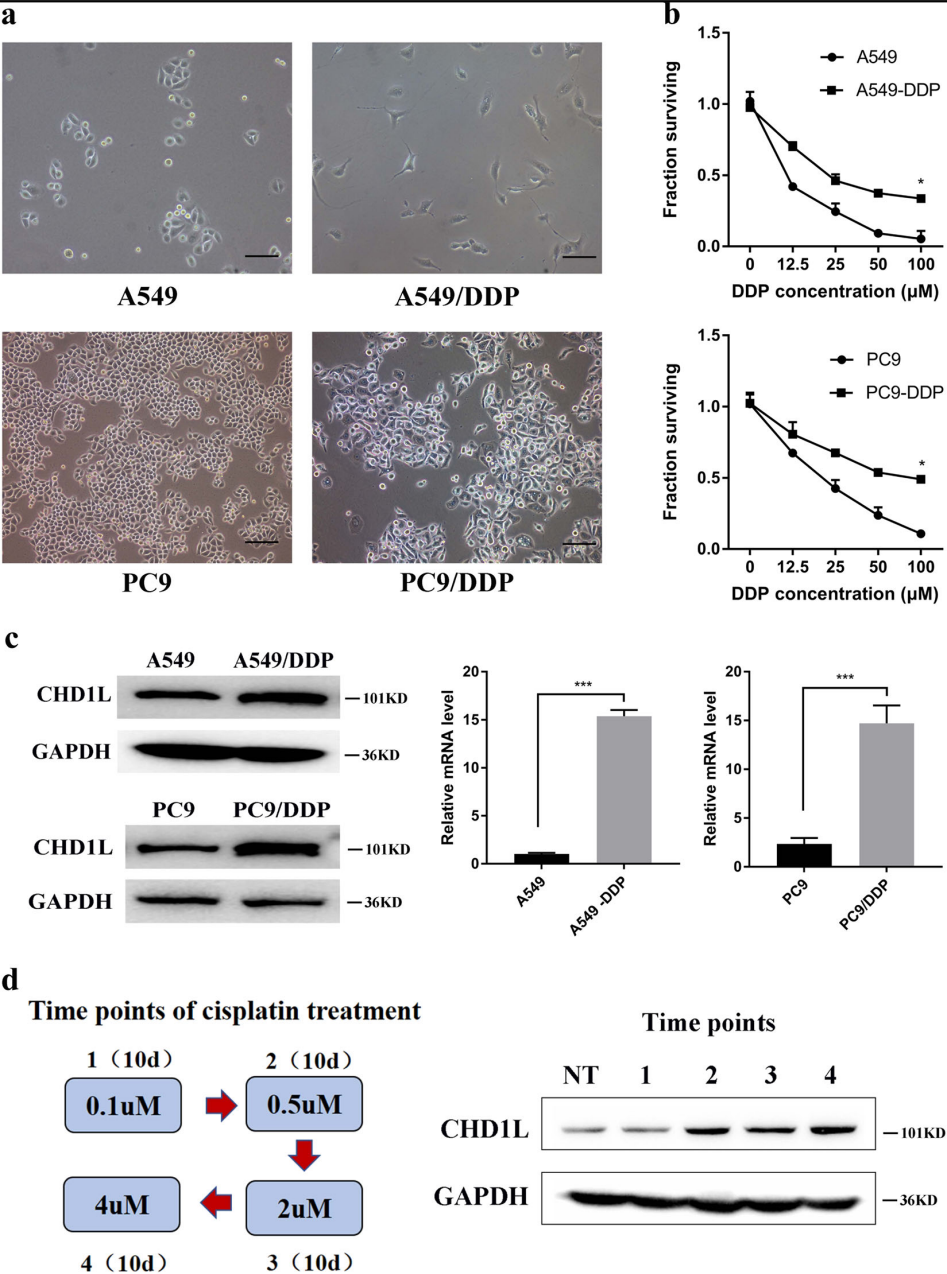
**Cisplatin resistance is associated with CHD1L activation.** **a** A549 and PC9 cells displayed epithelial morphology, and A549/DDP and PC9/DDP cells exhibited fibroblastic morphology (original magnification, ×200). **b** Two DDP-resistant cells and their parental cells were treated with indicated concentrations of cisplatin for 48 h and then were subjected to CCK assay (n = 5). The results show that A549/DDP and PC9/DDP cells are more resistant to cisplatin than their parental cells in vitro. **c** qRT-PCR and Western blotting illustrate increased expression of CHD1L in A549/DDP and PC9/DDP cells. **d** Schema of step-wise cisplatin treatment on PC9 cells; CHD1L expression during cisplatin treatment was measured using western blotting. Statistics were generated from three independent experiments. *P<0.05, **P<0.01, ***P<0.001, Student’s t-test; error bar: ±S.D

In order to validate the correlation between CHD1L and drug resistance in NSCLC cells, we constructed cisplatin-resistant A549 and PC9 cell lines that displayed distinctive epithelial morphology. The A549/DDP and PC9/DDP cells exhibited fibroblastic morphology (Fig. 3a). The CCK8 assay showed that both A549/DDP and PC9/DDP cells exhibited significantly higher resistance to cisplatin than non-DDP-resistant cells (Fig. 3b). QPCR and western blotting revealed that DDP-resistant cells had higher levels of CHD1L expression than their non-DDP-resistant cell controls (Fig. 3c). To investigate the correlation between CHD1L expression and cisplatin sensitivity, we treated PC9 cells with stepwise cisplatin to generate the cells bearing different extents of resistance. After treatment with the indicated concentration of cisplatin, the surviving cells were harvested at every 10 days to evaluate CHD1L expression. As shown in Fig. 3d, the expression of CHD1L in viable cells that sustained the escalating dosage of cisplatin was increased clearly at the second time point, and maintained its higher expression level in the following time point, which supporting our hypothesis that CHD1L is associated with cisplatin resistance (Fig. 3d).

### CHD1L suppresses cisplatin-induced apoptosis in cisplatin-resistance cells

**Fig. 4:**
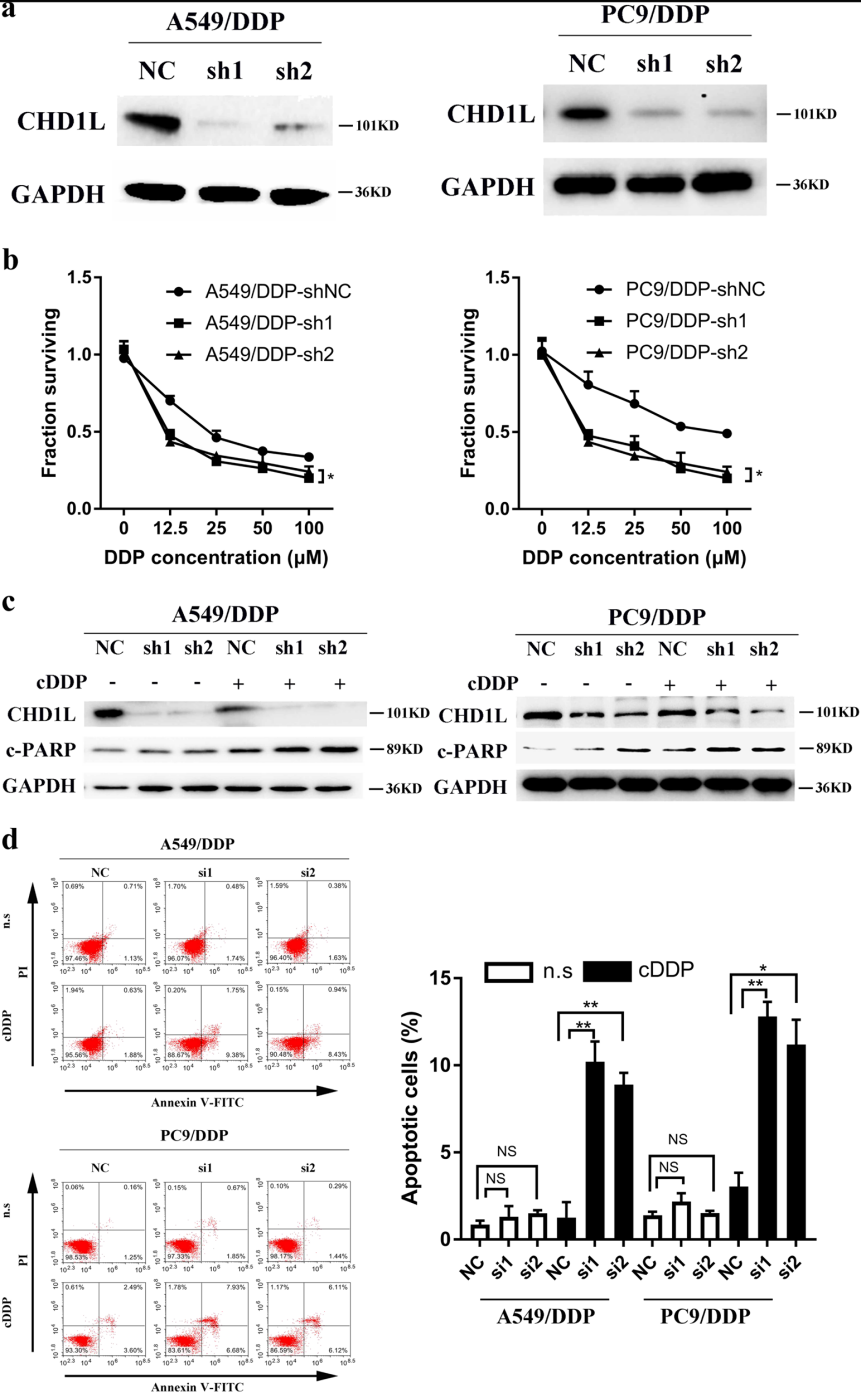
**CHD1L suppresses cisplatin-induced apoptosis in cisplatin-resistance cells.** **a** Western blotting reveals that CHD1L was efficiently knocked down by the treatment of CHD1L-shRNA-1 or CHD1L-shRNA-2 in A549/DDP and PC9/DDP cells. **b** A549/DDP and PC9/DDP cells were transfected with CHD1L-shRNA-1 or CHD1L-shRNA-2 and seeded in 96-well cell culture plates. The next day, cells were incubated with or without the indicated concentration of cisplatin for 48 h and subsequently subjected to a CCK assay. **c** WB assays (c-PARP, cleaved PARP) **d** Annexin-V-FITC/PI dual staining assay (left, representative plots for flow cytometry; right, bar charts indicating the average percentages of apoptotic cells). NC, negative control siRNA; si1, si2, CHD1L siRNAs; n.s, normal saline. *P<0.05; **P<0.01; NS, no significance

To assess the contribution of CHD1L in cisplatin resistance, two short hairpin RNAs (shRNAs) were generated and used to suppress CHD1L expression in two DDP-resistant cells with endogenous expression of CHD1L. The efficiency of CHD1L downregulation at the protein levels was evaluated by using western blotting (Fig. 4a). Knocking down of CHD1L effectively reduced the IC50 of cisplatin in both A549/DDP and PC9/DDP cells (Fig. 4b). Cisplatin accomplishes its anticancer effects by forming platinum-DNA adducts, which results in DNA damage and consequent apoptosis. We therefore hypothesized that CHD1L might inhibit cisplatin-induced apoptosis in NSCLC cells, resulting in cisplatin resistance. Supporting this hypothesis, knocking down CHD1L increased the cleavaged PARP in A549/DDP and PC9/DDP cells after cisplatin treatment (Fig. 4c). Annexin-V/propidium iodide (PI) staining revealed that dampening CHD1L expression significantly enhanced cisplatin-induced apoptosis in A549/DDP and PC9/DDP cells (Fig. 4d). To further understand the role of CHD1L in cisplatin resistance, we performed a competition assay in which normal A549/DDP cells (GFP-) were mixed with the GFP-labeled, shRNA-transfected cells at a 1:1 ratio and then treated with cisplatin. Excitingly, after cisplatin treatment the cells with endogenous levels of CHD1L became the dominant population (Supplementary Fig. 1c), indicating that CHD1L contributes to cisplatin resistance and suppression of cisplatin-induced apoptosis in NSCLC cells.

### Depletion of CHD1L enhances the sensitivity of xenograft tumors to cisplatin

**Fig. 5:**
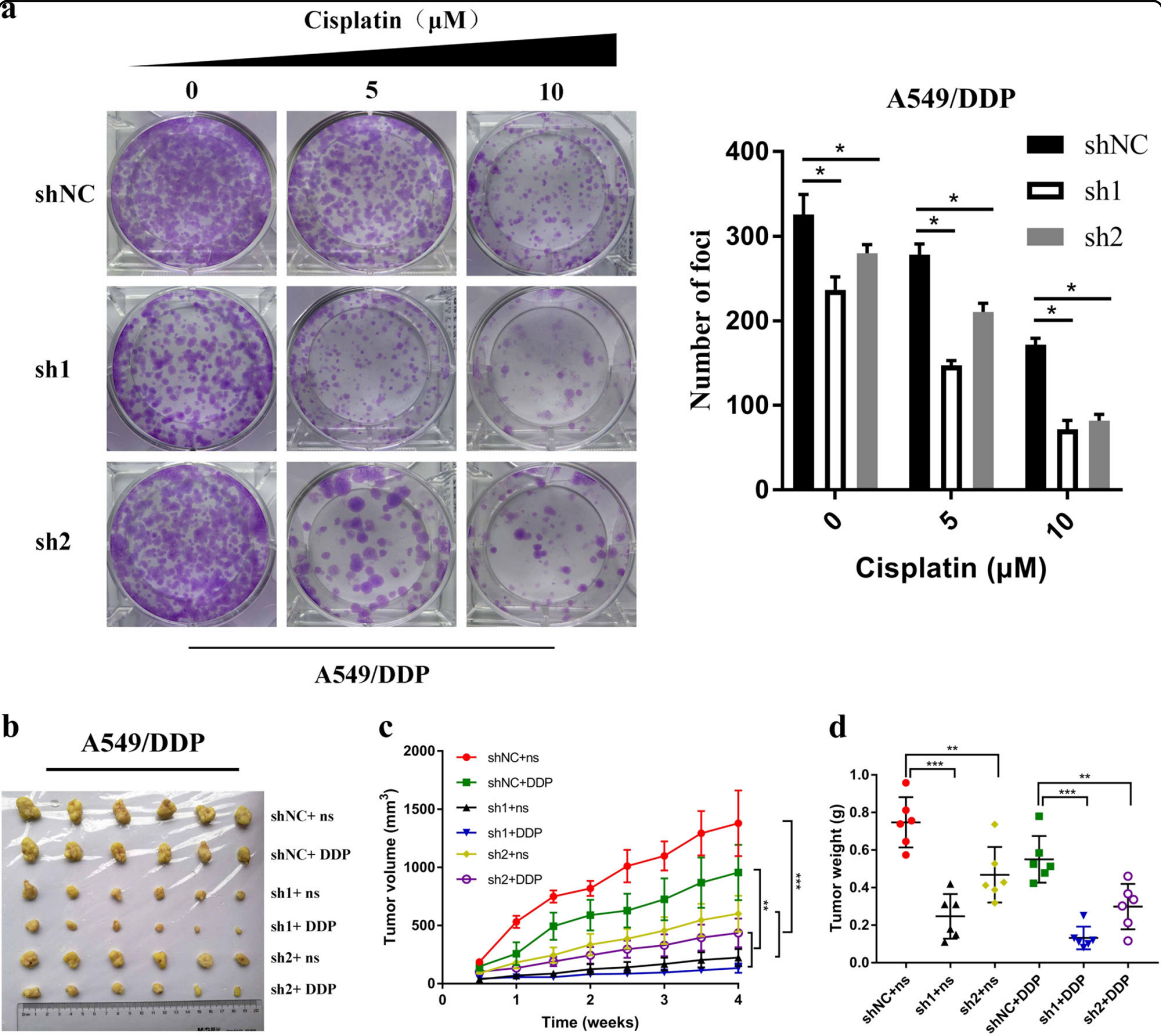
**Depletion of CHD1L expression enhances the sensitivity of xenograft tumors to cisplatin.** **a** A549/DDP cells transfected with CHD1L-shRNA-1 or CHD1L-shRNA-2 were treated with cisplatin at the indicated concentration for 14 days. Colonies were stained with crystal violet (Left). The number of colonies were taken from three independent experiments (Right). **b** CHD1L knockdown reverses cisplatin resistance of cisplatin-resistant cells in xenograft tumors implanted onto adult female nude mice. A549/DDP cells transfected with CHD1L-shRNA-1 or CHD1L-shRNA-2 were subcutaneously injected to generate xenograft tumors in nude mice; cisplatin treatment was performed as described in Materials and Methods. Normal saline (n.s.) was used as a treatment control. Images of xenograft tumors harvested at the end of the experiment. **c** Growth curves of tumor xenografts. **d** The weights of tumors are presented as a Cleveland dot plot, and the average ±S.D is included (n=6/group; **P< 0.01; ***P <0.001; NS, no significance)

To determine whether or not ectopic expression of CHD1L could decrease oncogenicity function of NSCLC cells, we used an A549/DDP cell line, which stably knocks down expression of CHD1L. In clonogenic assays, we found that silencing of CHD1L in combination with cisplatin treatment caused a marked inhibition of proliferation in A549/DDP cells (Fig. 5a). We next wanted to know if silencing of CHD1L can also tumors to cisplatin in vivo. To test this, we stably knocked down the expression of CHD1L in A549/DDP cells (A549/DDP-sh1 and A549/DDP-sh2; A549/DDP-shNC as negative control cells) and established subcutaneous xenografts in nude mice, which were then treated with cisplatin. Transfection of CHD1L shRNAs effectively inhibited the growth of NSCLC xenografts in nude mice. With the treatment of cisplatin, both the tumor volume and tumor weight were found to be reduced significantly (Fig. 5b–d). All these data indicate that CHD1L knockdown enhances the sensitivity of xenograft tumors to cisplatin treatment.

### ABCB1 is responsible for CHD1L-induced NSCLC cell cisplatin resistance

**Fig. 6:**
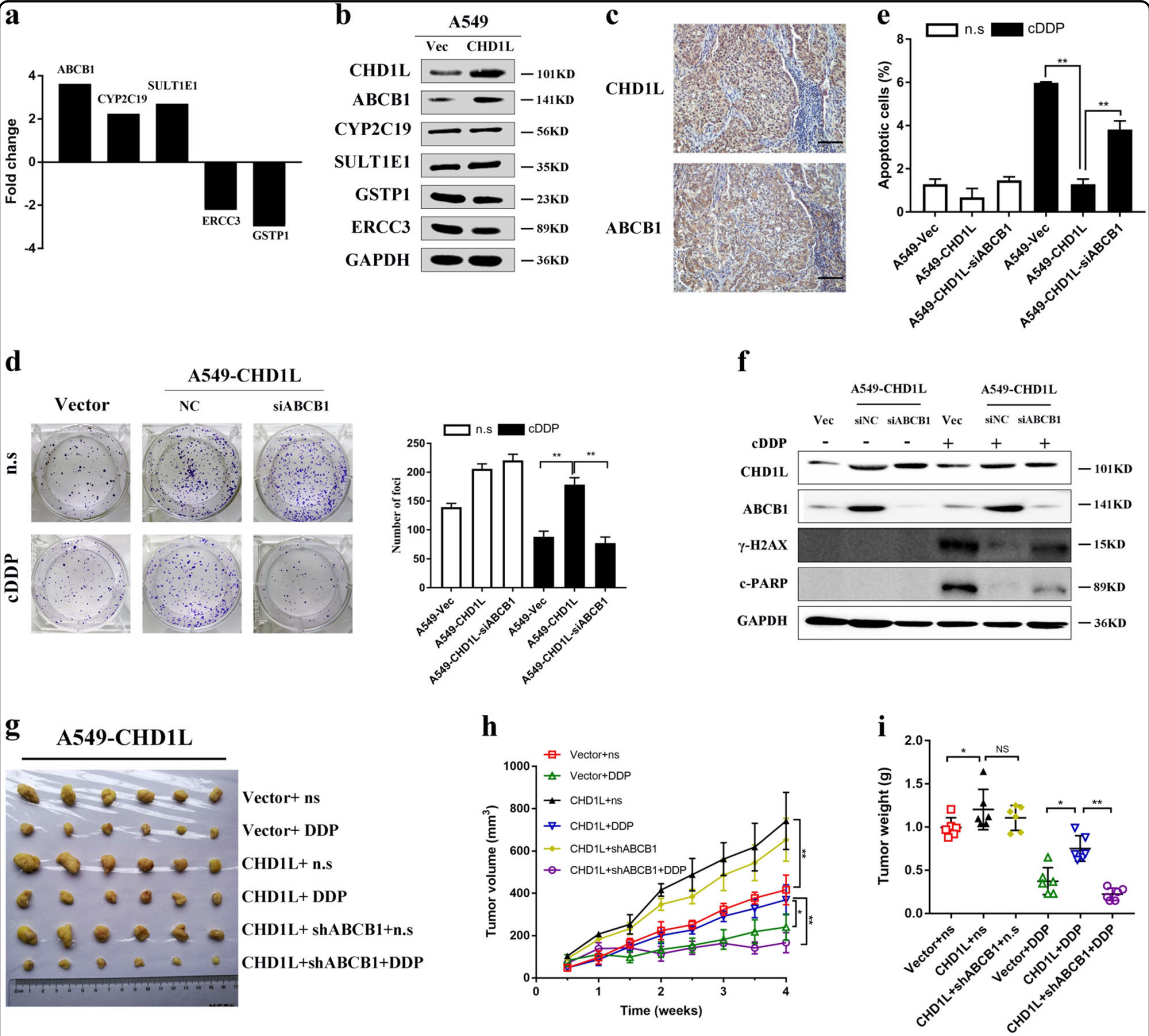
**ABCB1 is responsible for CHD1L-induced NSCLC cell cisplatin resistance.** **a** Five genes, *ABCB1*, *CYP2C19*, *SULT1E1*, *ERCC3*, and *GSTP1* were found to have at least a 2-fold mRNA differential expression in A549-CHD1L cells compared to that in A549-vec using Cancer Drug Resistance RT 2 Profiler™ PCR Array. **b** Expression of ABCB1, CYP2C19, SULT1E1, ERCC3, and GSTP1 verified in A549-CHD1L and respective control by western blot. **c** Overexpression of CHD1L and ABCB1 was examined by immunohistochemistry in NSCLC tissues; scale bar, 10 μm, original magnification, ×200. **d** Silencing of ABCB1 in combination with cisplatin caused a marked inhibition of proliferation in A549-CHD1L cells. **e** Annexin-V-FITC/PI dual staining assay show that the enhanced cisplatin-resistance ability in A549-CHD1L cells was inhibited by silencing of ABCB1. **f** Western blot analysis showed that the cisplatin induced γ-H2AX over-expression could be rescued by silencing ABCB1 in CHD1L-overexpressing NSCLC cells. **g** Images of xenograft tumors harvested at the end of the experiment. **h** Growth curves of tumor xenografts. **i** The weights of tumors are presented as a Cleveland dot plot, and the average± S.D. is included (n=6/group; **P<0.01; ***P<0.001; NS, no significance)

In order to determine any possible downstream targets of CHD1L in NSCLC cell cisplatin resistance, we analyzed mRNA expression of A549-CHD1L cells and its vector control, using Cancer Drug Resistance Real-time PCR Array containing 84 cell drug resistance-related genes. As shown in Fig. 6a, three upregulated genes (*ABCB1*, *CYP2C19*, and *SULT1E1*) and two downregulated genes (*ERCC3* and *GSTP1*), were identified by more than twofold change in mRNA levels between the two groups (Fig. 6a, Table 2). All candidate genes were further validated using immunoblotting. Only the blot of ATP-Binding Cassette Sub-Family B Member (ABCB1) was matched with the results from RT-PCR Array, therefore we pursued this candidate further (Fig. 6b). ABCB1 encoding P-glycoprotein (P-gp), is one of pharmaceutical carriers that can decrease the effective intracellular concentration of the drug, leading to drug resistance. In addition, a significant positive correlation between the overexpression of CHD1L and ABCB1 was evaluated in our large cohort of NSCLC tissues (Fig. 6c, *P* = 0.03, Supplementary Table 1). To clarify its role in CHD1L-induced cisplatin resistance, RNAi was used to knock down ABCB1 in A549-CHD1L cells. In clonogenic assays, we also found that silencing of ABCB1 in combination with cisplatin caused a marked inhibition of proliferation in A549-CHD1L cells (Fig. 6d). Annexin-V and PI staining revealed that silencing ABCB1 expression significantly enhanced cisplatin-induced apoptosis in A549-CHD1L cells (Fig. 6e). Histone H2AX phosphorylation at serine-139 (also known as γH2AX), is an established marker of DNA double-strand breaks. Our results showed that the cisplatin-induced γ-H2AX overexpression could be rescued by silencing ABCB1 in CHD1L-overexpressing NSCLC cells (Fig. 6f). To investigate whether ABCB1 is required for CHD1L-induced NSCLC cell cisplatin resistance, we established subcutaneous xenografts in female, adult, nude mice, which were then treated with cisplatin. Transfection of ABCB1 shRNAs showed no obvious inhibitory effects of NSCLC xenografts in nude mice (Fig. 6g), however, both the volume and tumor weight of the subcutaneous transplanted tumor decreased significantly with cisplatin treatment (Fig. 6h, i). Taken together, these data provided evidence that ABCB1 a downstream target of CHD1L-induced cisplatin resistance in NSCLC cells.

**Table 2:** List of genes differentially expressed in A549 cells after CHD1L overexpression using a Cancer Drug Resistance Real-time PCR Array

Gene	Fold change	Location	Function
Upregulated genes			
* ABCB1*	+3.63	7q21.12	Decreases drug accumulation
* ABCC1*	+1.25	16p13.11	Involves in multi-drug resistance
* ABCC2*	+1.00	10q24.2	Involves in multi-drug resistance
* ABCC3*	+1.02	17q21.33	Involves in multi-drug resistance
* ABCC5*	+1.01	3q27.1	Provides resistance to thiopurine anticancer drugs
* ABCG2*	+1.69	4q22.1	Responses to mitoxantrone and anthracycline exposure
* ACTB*	+1.16	7p22.1	Involves in cell motility, structure, integrity, and intercellular signaling
* AHR*	+1.45	7p21.1	Regulate xenobiotic-metabolizing enzymes
* AR*	+2.03	Xq12	Encode polyglutamine and polyglycine tracts
* ATM*	+1.01	11q22.3	Cell response to DNA damage
* B2M*	+1.14	15q21.1	Association with the MHC
* BCL2L1*	+1.14	20q11.21	Apoptotic inhibitor
* BLMH*	+1.02	17q11.2	Metabolic inactivation
* BRCA1*	+1.13	17q21.31	Maintains genomic stability
* BRCA2*	+1.62	13q13.1	Involves in maintenance of genome stability
* CCND1*	+1.12	11q13.3	Required for cell cycle G1/S transition
* CCNE1*	+1.13	19q12	Required for cell cycle G1/S transition
* CDK2*	+1.29	12q13.2	Regulates progression through the cell cycle
* CDK4*	+1.02	12q14.1	Regulates progression through the cell cycle
* CLPTM1L*	+1.67	5p15.33	Increases susceptibility to cancers
* CYP2C19*	+2.24	10q23.33	Variable ability to metabolize mephenytoin
* CYP2C8*	+1.53	10q23.33	Anticonvulsive drug mephenytoin
* CYP2E1*	+2.02	10q26.3	Involves in drug metabolism
* CYP3A5*	+1.46	18q21.1	Involves in drug metabolism
* DHFR*	+1.00	5q14.1	Identified on separate chromosomes
* EGFR*	+1.10	7p11.2	Promotes cell proliferation
* ELK1*	+1.25	Xp11.23	Promotes cell proliferation
* ESR1*	+1.07	6q25.1-q25.2	A ligand-activated transcription
* ESR2*	+1.49	14q23.2-q23.3	Inhibits cell proliferation
* FGF2*	+1.27	4q28.1	Nervous system development, wound healing, and tumor growth
* FOS*	+1.26	14q24.3	Cell proliferation and transformation
* GSTP1*	+1.02	11q13.2	Reduces glutathione and detoxification
* HPRT1*	+1.05	Xq26.2-q26.3	Plays a central role in the generation of purine nucleotides
* MET*	+1.40	7q31.2	Induces dimerization and activation of the receptor,
* MSH2*	+1.18	2p21-p16.3	Consistent with the characteristic alterations
* MYC*	+1.31	8q24.21	Participates in cell cycle progression, apoptosis and cellular transformation
* NAT2*	+1.19	8p22.	Associated with higher incidences of cancer and drug toxicity
* NFKB2*	+1.03	10q24.32	Involves in inflammation and immune function
* PPARA*	+1.27	22q13.31	Promotes proliferation
* PPARG*	+1.74	3p25.2	A regulator of adipocyte differentiation
* RARB*	+1.64	3p24.2	Mediates signaling in cell growth and differentiation
* SULT1E1*	+2.11	4q13.3	Controls levels of estrogen receptors
* TNFRSF11A*	+1.00	18q21.33	Promotes proliferation
* UGCG*	+1.41	9q31.3	The core structure of many glycosphingolipids
Downregulated genes			
* XPA*	−1.00	9q22.33	Plays a central role in nucleotide excision repair
* ABCC1*	−1.27	16p13.11	Involves in multi-drug resistance
* AP1S1*	−1.09	7q22.1	Involves in endocytosis and Golgi processing
* APC*	−1.19	5q22.2	Cell migration and adhesion
* ARNT*	−1.05	1q21.3	Involves in xenobiotic metabolism
* BAX*	−1.43	19q13.33	Involves in P53-mediated apoptosis
* BCL2*	−1.14	18q21.33	In multiple transcript variants
* CDKN1A*	−1.10	6p21.2	Interact with proliferating cell nuclear antigen,
* CDKN1B*	−1.15	12p13.1	Controls the cell cycle progression
* CDKN2A*	−1.13	9p21.3	Inhibits proliferation
* CDKN2D*	−1.09	19p13.2	Participates in proliferation
* CYP1A1*	−1.13	15q24.1	Involves in drug metabolism
* CYP1A2*	−1.02	15q24.1	Involves in drug metabolism
* CYP2B6*	−1.02	19q13.2	Involves in drug metabolism
* CYP2C9*	−1.23	10q23.33	Involves in drug metabolism
* CYP2D6*	−1.06	22q13.2	Involves in drug metabolism
* CYP3A4*	−1.04	7q22.1	Involves in drug metabolism
* EPHX1*	−1.25	1q42.12	Activation and detoxification of epoxides
* ERBB2*	−1.27	17q12	Promotes cell proliferation
* ERBB3*	−1.30	12q13.2	Promotes cell proliferation
* ERBB4*	−1.23	2q34	Promotes cell proliferation
* ERCC3*	−2.10	2q14.3	Nucleotide excision repair
* GAPDH*	−1.08	Xq26.2-q26.3	Identified as a moonlighting protein
* GSK3A*	−1.07	19q13.2	Regulates glycogen synthase and transcription factors
* GSTP1*	−2.86	11q13.2	Reduces glutathione and detoxification
* HIF1A*	−1.24	14q23.2	Involves in energy metabolism, angiogenesis, apoptosis
* IGF1R*	−1.21	15q26.3	Enhances cell survival
* IGF2R*	−1.12	6q25.3	Enhances cell survival
* MVP*	−1.47	16p11.2	Participates in multiple cellular processes
* NFKB1*	−1.02	4q24	Leads to cell development or delayed cell growth
* NFKBIB*	−1.07	19q13.2	A transcription factor
* NFKBIE*	−1.09	6p21.1	Promotes proliferation
* PPARD*	−1.89	6p21.31	Inhibits the ligand-induced transcriptional activity
* RARA*	−2.78	17q21.2	Implicated in regulation of development, differentiation, apoptosis
* RARG*	−1.23	12q13.13	Involves in various biological processes
* RB1*	−1.40	13q14.2	A negative regulator of the cell cycle
* RELB*	−1.02	19q13.32	Activation in anti-inflammatory decidual endothelial cells
* RPLP0*	−1.27	12q24.23	The functional equivalent of the *E. coli* L10 ribosomal protein
* RXRA*	−1.71	9q34.2	Mediates the biological effects of retinoids
* RXRB*	−1.19	6p21.32	Increases DNA binding
* SOD1*	−1.10	21q22.11	A homodimer to convert naturally occuring
* TOP1*	−1.28	20q12	Controls the topologic states of DNA during transcription.
* TOP2A*	−1.11	17q21.2	Controls the topologic states of DNA during transcription.
* TOP2B*	−1.32	3p24.2	Controls the topologic states of DNA during transcription.
* TP53*	−1.08	17p13.1	Participates in cell cycle arrest, apoptosis, senescence, DNA repair
* TPMT*	−1.19	6p22.3	Correlated with variations in sensitivity and toxicity
* XPC*	−1.02	3p25.1	Plays an important role in the early steps of global genome nucleotide excision repair

### ABCB1 upregulation by CHD1L was partly dependent on c-Jun

**Fig. 7:**
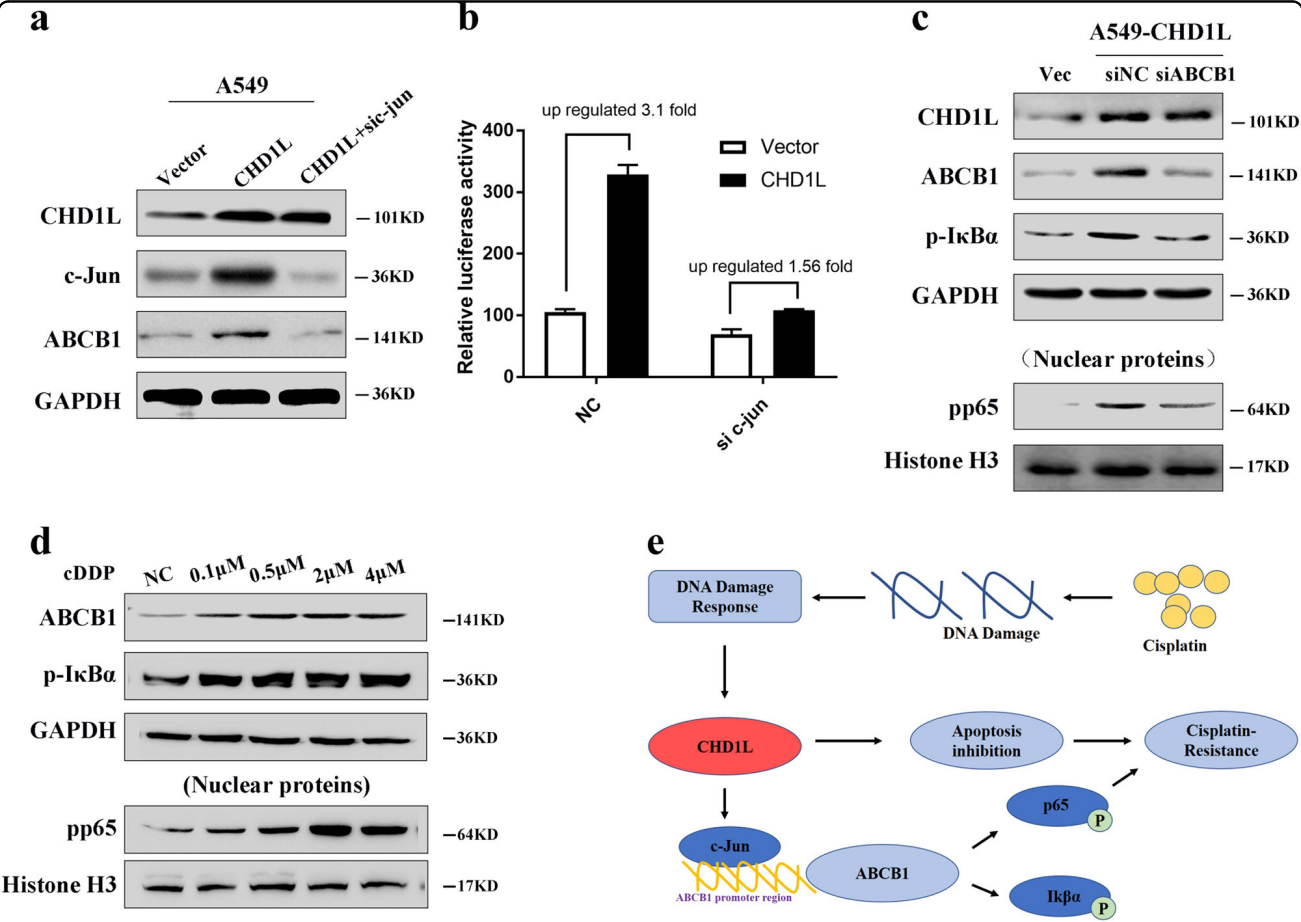
**ABCB1 upregulation by CHD1L was partly dependent on c-Jun.** **a** Western blot analysis showed that both c-Jun and ABCB1 were increased by enforced CHD1L expression in A549 cells. **b** Dual luciferase reporter assays show that the increased transcriptional activity and expression levels of ABCB1 by CHD1L were largely blocked after silencing c-Jun in CHD1L-overexpressing NSCLC cells. **c** The effects on the expression of CHD1L, phosphorylated p65 and phosphorylation of IκBα were analyzed by western blot. Scramble siRNA (siNC) was used as control. **d** ABCB1 expression and NF-κb activation during cisplatin as depicted in panel. **e** Schematic diagram depicting a proposed model for a major mechanism of CHD1L and its upregulation in the promotion of NSCLC cell cisplatin-resistance

Several studies reported that c-Jun could bind to ABCB1 and promote its transcription. Our Western blot analysis showed that both c-Jun and ABCB1 were increased by enforced CHD1L expression in A549 cells (Fig. 7a). Moreover, the dual luciferase reporter assays demonstrated that the increased transcriptional activity and expression of ABCB1 by CHD1L were largely blocked after silencing c-Jun in CHD1L-overexpressing NSCLC cells (Fig. 7b). Meanwhile, silencing ABCB1 in CHD1L-overexpressed NSCLC cells could increase the phosphorylation of p65 and IκBα. (Fig. 7c). Through the concentration gradient experiment, we proved that the expression level of ABCB1 in PC9 cells is consistent with cisplatin gradient, and the NFκB downstream IκBα and p65 was phosphoried with the upregulated ABCB1 (Fig. 7d). Schematic diagram depicting a proposed model for a major mechanism of CHD1L and its upregulation in the promotion of NSCLC cell cisplatin-resistance. Cisplatin generates DNA damage, which eventually leads to cell apoptosis. Meanwhile, CHD1L is upregulated after cisplatin treatment, possibly through a direct or indirect induction by DNA damage response. Therefore, CHD1L functions through activating NF-κB signaling to mediate resistance to cisplatin (Fig. 7e).

## Discussion

Over amplification of chromosome 1q has been found to play an important role in tumor pathogenesis and disease progression in several human cancers; including NSCLC. Several candidate oncogenes including CHD1L and MUC1 have been found on chromosome region 1q21. These candidate oncogenes have been found to be associated with a poor chemotherapy response and poor prognosis in HCC and ovarian cancer, respectively, implying that putative oncogenes within this region may contribute to not only tumor progression but also to chemotherapy resistance in human cancers. Previously we found patients with NSCLC had a significantly increased frequency of overexpression and amplification of CHD1L compared to healthy lung tissue. Overexpression of CHD1L was associated with advanced disease stage and overall poorer outcomes in NSCLC patients that were treated with radical surgery. In the present study, we further showed that overexpression of CHD1L was associated with a poor chemotherapy response and worse outcome in cisplatin-treated NSCLC patients. These data, taking together, suggest that CHD1L might have an important role in cisplatin resistance and might serve as a predictor of chemotherapy response in NSCLC.

Consistent with our clinical observations, we found that overexpression of CHD1L desensitized NSCLC cells to cisplatin, and conversely, downregulation of CHD1L increased the sensitivity to cisplatin. These results indicate that CHD1L may cause the development of cisplatin resistance known to acquire malignant phenotypes after repeated exposure to cisplatin^18^. The primary anticancer mechanism of cisplatin is to bind DNA covalently to form platinum-DNA adducts and induce DNA damage in the proliferating cancer cells^19^. In recent years, CHD1L has been defined as a DNA damage–response protein, and could be recruited to the damaged sites by associated factor PARP-1^20^. Cisplatin-DNA platinum adducts may function as an activator of some unknown molecules which is effective to upregulate CHD1L, or there may be some other complicated mechanisms which need to be further investigate. Interestingly, we also found that cisplatin treatment increased the expression level of CHD1L in PC9 cells, and it is possible this increased expression of CHD1L contributes to the resistant cell subtype transformation. We called this process of cisplatin treatment demonstrating an initially favorable outcome, then later on developing chemoresistance “acquired resistance”. Our results suggest that silencing CHD1L expression may have therapeutic potential, especially in enhancing the chemosensitivity of NSCLC to cisplatin. Our results provide us with new insights into the role CHD1L in drug resistance, and importantly, CHD1L may act as a potential target to overcome cisplatin resistance in NSCLC.

The molecular mechanism by which CHD1L regulates cancer cell cisplatin resistance remains unclear. In our recent study, we found that CHD1L might confer chemoresistance by inhibiting the Nur77/ Cyto c/caspase 9 pathway in HCC^10^. However, in this study we did not look for altered levels of active Nur77, Cyto c and caspase9 before and after CHD1L overexpression or knockdown. Therefore, we concluded that in our NSCLC cells, CHD1L regulated cancer cell cisplatin resistance by the regulation of targets and/or pathways other than the activation of Nur77/Cyto c/caspase 9, suggesting that the mechanism(s) by which CHD1L regulates cancer cisplatin resistance may be tumor-type specific.

To better understand the potential downstream molecular mechanism of CHD1L in drug resistance, a Cancer Drug Resistance Real-time PCR Array containing 84 well-known drug resistance-related genes was used to compare mRNA expression profiles between A549-CHD1L cells and those of control A549-vector cells. Of the 84 well-known resistance genes, five were found to be differentially expressed (mRNA levels altered by two fold or more). *ABCB1*, *CYP2C19*, and *SULT1E1* were all upregulated and *ERCC3* and *GSTP1* were downregulated. Western blot data indicate both ABCB1 and ERCC3 are consistent to the result, however, downregulated CHD1L in A549-DDP cells decrease the ABCB1 and ERCC3, indicating that ERCC3 may be regulated more complicated than ABCB1 do. As a result, we just focus on ABCB1 in present study. (supplementary Fig. 2a). ABCB1, initially isolated in drug-resistant Chinese hamster ovary cancer cells^21^, was hypothesized to be the most obvious choice for a downstream target gene of CHD1L in NSCLC cells. And indeed, we did observe a significant positive correlation between the overexpression of CHD1L and ABCB1 in our large cohort of NSCLC tissues. These results, collectively, suggest that in NSCLC cells, CHD1L might regulate cell cisplatin resistance by the regulation of ABCB1.

In recent years, numerous studies have shown that ABCB1 is widely expressed in human tumor cells at different stages^22^. The patients who suffer from tumors with high levels of ABCB1, including patients with colorectal cancer^23^, pancreatic cancer^24^, liver cancer^25^, adrenal cortex carcinoma^26^, acute leukemia^27^, and ovarian cancer^28^, are usually found to also have a poorer prognosis. It is also reported that ABCB1 has an important effect on absorption, distribution, metabolism, and excretion of its substrate drugs^29^. Inhibition of ABCB1 efflux activity increases the accumulation of chemotherapeutic drugs in tumor cells with high expression of ABCB1, thereby enhancing the inhibitory effect of chemotherapeutic drugs on tumor cells^30^. The results of our rescue experiment indicate that CHD1L-mediated cisplatin-resistance can be dramatically prevented by knockdown of ABCB1. These data suggest that ABCB1 might be a critical downstream target of CHD1L and may be responsible for the CHD1L-induced cisplatin-resistance in NSCLC cells.

To date, however, the mechanisms by which CHD1L regulates ABCB1 expression have not been elucidated. Our previous study found no evidence to support a direct binding of CHD1L on the promoter region of ABCB1, indicating that an indirect regulatory mechanism might exist between CHD1L and ABCB1 in NSCLCs. Because it has been improved that c-Jun could bind to ABCB1 promoter^31^, we therefore verified whether c-Jun expression could be affected by CHD1L. The Western blot analysis showed that both c-Jun and ABCB1 were increased by enforced CHD1L expression in A549 cells. Moreover, ABCB1 expression depends on c-Jun level. The dual luciferase reporter assay for ABCB1 showed that overexpressed CHD1L increase the luciferase activity of ABCB1, while, blocking c-Jun by siRNA approach significantly attenuates the luciferase activity, indicating that c-Jun is involved in the promoting effect of CHD1L on ABCB1 expression. Furthermore, the functional studies also showed that CHD1L-induced cisplatin-resistance could be attenuated by c-Jun down-regulation in A549 cells. All together, our results indicated that the c-Jun mediated the promoting effect of CHD1L on ABCB1 expression.

NF-κB signaling is a well-known survival-related pathway that upregulates anti-apoptotic genes, and has recently been assessed to inhibit apoptosis and mediate cisplatin resistance and tumor development in cancer^32–34^. A recent study reported that NF-κB pathways was involved in the development of multiple drugs resistance (MDR) in MCF-7/ADR cells through the upregulation of the ABCB1 gene expression^35^. We thus verified the correlation between ABCB1 expression and NF-κB signaling activation in CHD1L overexpressed lung cancer cell lines. Through the concentration gradient experiment, we proved that the expression level of ABCB1 in A549 cells is consistent with cisplatin gradient, and the NFκB downstream IκBα and p65 was phosphoried with the upregulated ABCB1. Besides, silencing ABCB1 in CHD1L ectopic expressed A549 cells will decrease the phosphorylation of p65 and IκBα. However, we failed to prove the direct binding between ABCB1 and NFκB promoter region, but the results could indicate that the NF-κB pathway is closely associated with ABCB1 expression.

In summary, our study describes, for the first time, that CHD1L contributes to cisplatin resistance in NSCLC. Furthermore, we demonstrated that CHD1L plays a critical role in inducing cisplatin-resistance of NSCLC cells via upregulation of ABCB1 through c-Jun. Our results suggest that CHD1L and ABCB1 may serve as potential therapeutic targets to overcome cisplatin-resistance in NSCLC.

## Supplementary information

Supplementary Figure legends

Figure S1

Figure S2

Supplementary Table 1

## References

[1] Harpole DH (1995). Stage I nonsmall cell lung cancer. A multivariate analysis of treatment methods and patterns of recurrence. Cancer.

[2] Siegel RL, Miller KD, Jemal A (2018). Cancer statistics. CA Cancer J. Clin..

[3] Gridelli C (2015). Non-small-cell lung cancer. Nat. Rev. Dis. Prim..

[4] Grondin SC (2002). Current concepts in the staging of non-small cell lung cancer. Surg. Oncol..

[5] Tan XL (2011). Genetic variation predicting cisplatin cytotoxicity associated with overall survival in lung cancer patients receiving platinum-based chemotherapy. Clin. Cancer Res..

[6] Peron J (2014). An effective and well-tolerated strategy in recurrent and/or metastatic head and neck cancer: successive lines of active chemotherapeutic agents. BMC Cancer.

[7] Ma NF (2008). Isolation and characterization of a novel oncogene, amplified in liver cancer 1, within a commonly amplified region at 1q21 in hepatocellular carcinoma. Hepatology.

[8] Chen L (2010). CHD1L promotes hepatocellular carcinoma progression and metastasis in mice and is associated with these processes in human patients. J. Clin. Investig..

[9] Chen L (2010). Chromosome 1q21 amplification and oncogenes in hepatocellular carcinoma. Acta Pharmacol. Sin..

[10] Chen L (2011). Clinical significance of CHD1L in hepatocellular carcinoma and therapeutic potentials of virus-mediated CHD1L depletion. Gut.

[11] Cheng W (2013). CHD1L: a novel oncogene. Mol. Cancer.

[12] Wu J (2014). Presence of CHD1L over-expression is associated with aggressive tumor biology and is a novel prognostic biomarker for patient survival in human breast cancer. PLoS One.

[13] Su, Z. et al. CHD1L is a novel independent prognostic factor for gastric cancer. *Clin. Transl. Oncol.* **16**, 702–707 (2014). 10.1007/s12094-013-1136-8 24258459

[14] Ji X (2013). CHD1L promotes tumor progression and predicts survival in colorectal carcinoma. J. Surg. Res..

[15] Tian F (2013). Expression of CHD1L in bladder cancer and its influence on prognosis and survival. Tumour Biol..

[16] He WP (2012). CHD1L protein is overexpressed in human ovarian carcinomas and is a novel predictive biomarker for patients survival. BMC Cancer.

[17] He LR (2015). Overexpression of CHD1L is positively associated with metastasis of lung adenocarcinoma and predicts patients poor survival. Oncotarget.

[18] Wang H (2014). Acquisition of epithelial-mesenchymal transition phenotype and cancer stem cell-like properties in cisplatin-resistant lung cancer cells through AKT/beta-catenin/Snail signaling pathway. Eur. J. Pharmacol..

[19] Seve P, Dumontet C (2005). Chemoresistance in non-small cell lung cancer. Curr. Med. Chem. Anti-Cancer Agents.

[20] Ahel D (2009). Poly(ADP-ribose)-dependent regulation of DNA repair by the chromatin remodeling enzyme ALC1. Science.

[21] Panczyk M, Salagacka A, Mirowski M (2007). MDR1 (ABCB1) gene encoding glycoprotein P (P-gp), a member of ABC transporter superfamily: consequences for therapy and progression of neoplastic diseases. Post. Biochem..

[22] Wellhoner H (2012). Reversing ABCB1-mediated multi-drug resistance from within cells using translocating immune conjugates. J. Drug Target..

[23] Yuan ZT (2017). Bufalin reverses ABCB1-mediated drug resistance in colorectal cancer. Oncotarget.

[24] Harpstrite SE, Gu H, Natarajan R, Sharma V (2014). Interrogation of multidrug resistance (MDR1) P-glycoprotein (ABCB1) expression in human pancreatic carcinoma cells: correlation of 99mTc-Sestamibi uptake with western blot analysis. Nucl. Med. Commun..

[25] Yang T (2013). MiR-223 modulates multidrug resistance via downregulation of ABCB1 in hepatocellular carcinoma cells. Exp. Biol. Med..

[26] Theile D, Haefeli WE, Weiss J (2015). Effects of adrenolytic mitotane on drug elimination pathways assessed in vitro. Endocrine.

[27] Zhan L (2017). ABCB1 polymorphisms and childhood acute lymphoblastic leukemia risk: a meta-analysis. Crit. Rev. Eukaryot. Gene Expr..

[28] Bjorn N (2018). ABCB1 variation affects myelosuppression, progression-free survival and overall survival in paclitaxel/carboplatin-treated ovarian cancer patients. Basic Clin. Pharmacol. Toxicol..

[29] Mealey KL (2010). Oral bioavailability of P-glycoprotein substrate drugs do not differ between ABCB1-1Delta and ABCB1 wild type dogs. J. Vet. Pharmacol. Ther..

[30] Shi Z (2011). Sildenafil reverses ABCB1- and ABCG2-mediated chemotherapeutic drug resistance. Cancer Res..

[31] Liu M (2008). Modulation of multidrug resistance in cancer cells by the E3 ubiquitin ligase seven-in-absentia homologue 1. J. Pathol..

[32] Chock KL, Allison JM, Shimizu Y, ElShamy WM (2010). BRCA1-IRIS overexpression promotes cisplatin resistance in ovarian cancer cells. Cancer Res..

[33] Wu DW (2015). FHIT loss confers cisplatin resistance in lung cancer via the AKT/NF-kappaB/Slug-mediated PUMA reduction. Oncogene.

[34] Zhang J (2018). Attenuated TRAF3 fosters alternative activation of NF-kappaB and reduced expression of anti-viral interferon, TP53, and RB to promote HPV-positive head and neck cancers. Cancer Res..

[35] Wei Y (2016). PA-MSHA inhibits the growth of doxorubicin-resistant MCF-7/ADR human breast cancer cells by downregulating Nrf2/p62. Cancer Med..

