# Supplementary material for: CHD1L contributes to cisplatin resistance by upregulating the ABCB1–NF-κB axis in human non-small-cell lung cancer
Source: Cell Death Dis. 2019 Feb 4;10(2):99. doi: 10.1038/s41419-019-1371-1 (PMC6362241; doi:10.1038/s41419-019-1371-1)
Supplement: Supplementary file 1 — Supplementary Figure legends [file 41419_2019_1371_MOESM1_ESM.docx]

**Supplementary Figure legend**

**Figure 1**: **CHD1L suppresses cisplatin-induced apoptosis in NSCLC cells.** (a) A549 were transfected with CHD1L plasmid and seeded in 96-well cell culture plates. The next day, cells were incubated with or without the indicated concentration of cisplatin for 48 h and subsequently subjected to a CCK assay. (b) WB assay for c-PARP (cleaved PARP). (c) Fluorescent images of A549/DDP-shCTR-GFP and A549/DDP-shCHD1L-GFP cells mixed with A549 cells before and after cisplatin treatment. And Statistical analysis of flow cytometry data.

**Figure 2**: **ABCB1 is responsible for CHD1L-induced NSCLC cell cisplatin resistance.** (a) Expression of ERCC3 and GSTP1 verified in A549/DDP-sh1 and respective control by western blot. (b) ABCB1 expression in A549/DDP cells and A549/DDP -shCHD1L
