# Supplementary material for: CHD1L contributes to cisplatin resistance by upregulating the ABCB1–NF-κB axis in human non-small-cell lung cancer
Source: Cell Death Dis. 2019 Feb 4;10(2):99. doi: 10.1038/s41419-019-1371-1 (PMC6362241; doi:10.1038/s41419-019-1371-1)
Supplement: Supplementary file 4 — Supplementary Table 1 [file 41419_2019_1371_MOESM4_ESM.docx]

**Table 1** Association between expression of CHD1L and ABCB1 in NSCLC patients

|  |  | **CHD1L expression level** | | |
| --- | --- | --- | --- | --- |
| **Variable** | **cases** | **Normal expression** | **Overexpression** | ***P value*** |
| ABCB1 |  |  |  | 0.03 |
| Normal expression | 114 | 74 (62.2%) | 40(37.8%) |  |
| Overexpression | 119 | 61 (53.5%) | 58 (46.5%) |  |
|  |  |  |  |  |

* Chi-square test.

Abbreviation: CHD1L, chromodomain helicase/ATPase DNA binding protein 1-like gene; NSCLC, non-small-cell lung carcinoma; ABCB1, ATP-Binding Cassette Sub-Family B Member
